# DAPAR & ProStaR: software to perform statistical analyses in quantitative discovery proteomics

**DOI:** 10.1093/bioinformatics/btw580

**Published:** 2016-09-06

**Authors:** Samuel Wieczorek, Florence Combes, Cosmin Lazar, Quentin Giai Gianetto, Laurent Gatto, Alexia Dorffer, Anne-Marie Hesse, Yohann Couté, Myriam Ferro, Christophe Bruley, Thomas Burger

**Affiliations:** 1 Université Grenoble Alpes, BIG-BGE, Grenoble, France; 2 CEA, BIG-BGE, Grenoble, France; 3 INSERM, BGE, Grenoble, France; 4 Computational Proteomics Unit, Cambridge, UK; 5 Cambridge Center for Proteomics, Cambridge, UK; 6 CNRS, BIG-BGE, Grenoble, France

## Abstract

**Summary:**

DAPAR and ProStaR are software tools to perform the statistical analysis of label-free XIC-based quantitative discovery proteomics experiments. DAPAR contains procedures to filter, normalize, impute missing value, aggregate peptide intensities, perform null hypothesis significance tests and select the most likely differentially abundant proteins with a corresponding false discovery rate. ProStaR is a graphical user interface that allows friendly access to the DAPAR functionalities through a web browser.

**Availability and implementation:**

DAPAR and ProStaR are implemented in the R language and are available on the website of the Bioconductor project (http://www.bioconductor.org/). A complete tutorial and a toy dataset are accompanying the packages.

The objectives of quantitative discovery proteomics are to identify proteins in several biological samples that separate into at least two different biological conditions and to perform a relative quantification, so as to discriminate between the proteins which are significantly differentially abundant, and those which are not. This classically involves numerous steps: (i) protein extraction; (ii) proteins digestion into peptides; (iii) liquid chromatography and tandem mass spectrometry analysis; (iv) peptide identification on the basis of the fragmentation spectra; (v) peptide quantitation on the basis of the precursor chromatograms (XIC) and (vi) peptide aggregation into protein identity and abundance. The outcome of this analytical pipeline is a *quantitative dataset* that contains protein abundance across all replicates.

Once the quantitative dataset is available, the *quantitative analysis* may start. Its objective is to rely on an efficient and reproducible statistical pipeline to isolate the subset of proteins that are characteristic of the differences between the biological conditions, on which further more exhaustive wet-laboratory experiments will be performed.

Numerous tools are available to perform such quantitative analysis, either as stand-alone tools (e.g. MSstats; Choi *et al.*, 2014) or as a module of a larger bioinformatics tool (e.g. Skyline; MacLean *et al.*, 2010), or as generic software that is not restricted to proteomics, but can be used in a wider omics context (e.g. InfernoRDN—Former DAnTE; Polpitiya *et al.*, 2008) or even for general purpose statistics (e.g. JMP—http://www.jmp.com/). It is also possible to sort the available tools according to their code being open (MSstats and more generally any R package) or not (Perseus—http://www.biochem.mpg.de/5111810/perseus), as well as according to the presence of a graphical user interface (GUI) or not: generally most of the R packages are not fit with a GUI, while other software tools are. To date, the only software tool that is based on R and which is endowed with a GUI is InfernoRDN. However, the underlying R packages are not accessible, so that the code is not really open, and the GUI only works on Windows operating systems. As a result, to the best of the authors’ knowledge, there is so far no software tool that is (i) devoted to proteomics; (ii) devoted to quantitative analysis; (iii) with open-source code that guarantees reproducibility, interoperability and quality control of the code; (iv) with a user-friendly GUI and (v) which can be operated on any operating system. This lack has motivated the developments reported here. In general, quantitative analysis is composed of the following steps:

1. **Filtering:** Some peptides or proteins may be discarded, on the basis of several user-defined criteria (number of missing values within each or across all the biological condition(s), contaminant database, decoy sequences, etc.).
2. **Normalization:** The protein abundances are rescaled (within or between conditions) to account for the variability between the analyses. Several algorithms can be used: quantile normalization (Bolstad, 2007), abundance normalization, scaling/centering (either globally applied or by condition), etc.
3. **Imputation:** To maximize the power of the statistical analysis, the missing values are imputed. This is achieved with one of the multiple available algorithms that accounts in a specific manner each for the specific nature of missing values (missing at random, or lower abundance censorship): *k* Nearest Neighbors (Hastie *et al.*, 2001), Maximum Likelihood Estimation (Schafer, 2008), Bayesian Principal Component Analysis (Stacklies *et al.*, 2007), Quantile Regression to Impute Left-Censored data (Lazar, 2015), etc.
4. **Aggregation:** The peptide intensities are aggregated together so as to infer back the abundances of the proteins originally present in the samples. Several aggregation functions are classically used: sum, mean or median of the intensities of a set of peptides (all of them, the protein specific ones or only the *N* most abundant ones).
5. **Differential analysis:** Finally, null hypothesis significance testing (with a Welch or limma *t*-tests; Ritchie *et al.*, 2015), as well as *P*-value adjustment are conducted, leading to a list of differentially abundant proteins endowed with a false discovery rate estimation.

DAPAR (differential analysis of protein abundance with R) is an R package that either proposes new algorithms for these five computational steps or simply binds the R packages implementing pre-existing state-of-the-art methods (refer to the ProStaR and DAPAR tutorial for an updated list of the available algorithms). The main feature of DAPAR is to gather in a single package, all the necessary statistical routines for quantitative analysis. Moreover, it is completely compatible with (i) the MSnbase package (Gatto and Lilley, 2012), which provides a standard format for quantitative datasets, as well as with (ii) any bioconductor package, so that its functionalities can be easily extended. However, as is, its use requires being comfortable with R programming, which is not the case for all proteomics practitioners.

This is why DAPAR is accompanied by ProStaR, a package that relies on Shiny technology (http://shiny.rstudio.com/) to dynamically build web-based GUI to DAPAR functionalities. All the user has to do is to copy–paste the following command lines

source (‘http://www.bioconductor.org/biocLite.R’) biocLite (‘DAPAR’); biocLite (‘Prostar’); library (Prostar); Prostar ()

in the R console to open the GUI and to start the quantitative analysis by a series of clicks. Moreover, ProStaR is also available in server mode: a single (server) machine is installed and maintained with R, DAPAR and ProStaR, on which each practitioner connects through a given URL. This makes ProStaR particularly suited for proteomics labs where a single bioinformatician deploys and maintains the tools that are used by the proteomicians for their data analyses. In addition, to providing menus devoted to each of the five processing steps (filtering, normalization, imputation, aggregation and differential analysis), ProStaR provides import/export functionalities, as well as a ‘descriptive statistics’ menu where it is possible to visualize the dataset in hands, so as to best understand it or to produce display elements for publications.

The packages DAPAR and ProStaR are separated for two reasons: first, ProStaR may be bypassed by any R coder that may want to directly access the low level functions of DAPAR, script their own pipelines and reproduce them in a better and simpler way. Second, the DAPAR functions can be directly mapped to other GUI (such as for instance ProLine software—http://proline.profiproteomics.fr/), so as to provide the same statistical pipeline in a different computational environment.

DAPAR and ProStaR are actively maintained. Further versions of DAPAR will include additional algorithms for the five aforementioned processing steps, as well as possibly new steps, such as for instance, bioanaylsis and biological inference. ProStaR will include the interfaces to these new functionalities, as well as predefined pipelines proposing only a restricted set of functionalities that are particularly adapted to specific proteomics analysis (e.g. tandem affinity purification and subcellular localization). Finally, a demo version of ProStaR can be directly tested online at the following URL: http://www.prostar-proteomics.org.
